# Mitochondrial genome characterization and phylogenetic analysis of bird schistosome *Trichobilharzia szidati*

**DOI:** 10.1080/23802359.2020.1715299

**Published:** 2020-06-29

**Authors:** Xu Wang, Na Liu

**Affiliations:** College of Life Sciences, Henan Agricultural University, Zhengzhou, China

**Keywords:** *Trichobilharzia szidati*, mitochondrial genome, phylogenetic analyses

## Abstract

In the present study, the complete mitochondrial genome of *T. szidati* was assembled by next generation sequencing (NGS). We found that the complete mitochondrial genome of *T.szidati* is 14, 303 bp in length and consists of 3023 (21.1%) adenine, 1153 (8.1%) cytosine, 3432 (24.0%) guanosine and 6695 (46.8%) thymine. The genome contains 12 conserved core protein-coding genes (*atp6, cox1, cox2, cox3, nad1, nad2, nad3, nad4, nad4L, nad5, nad6, CYTB*), 21 tRNA genes, 2 rRNA genes and 1 D-loop region. Phylogenetic analysis showed that *T. szidati* has a close relationship with *T. regent.* Knowledge of mitochondrial genome of *T. szidati* could provide useful information for the further studies of evolutionary biology, epidemiology and species identification.

*Trichobilharzia* is a kind of thread-like schistosome and it is distributed in birds worldwide (Yakhchali et al. 2016). It was reported that there were over 30 species in this kind of schistosomes (Loker 2009). *T. szidati* is one of the species that belongs to Schistosomatidae, Trematoda (Brant and Loker 2009). *T. szidati* can only parasitize in specific birds hosts whereas its larvae could penetrate, transform and migrate in all kinds of hosts (Le et al. 2002). In freshwater, the larva of *T. szidati* has the ability to penetrate human skin. Besides, it may cause human cercarial dermatitis (Martin Kašný 2011). Adult worms of *Trichobilharzia* cause extensive damage as they migrate and lay larvae in their preferred site (either the nasal mucosa or visceral capillaries) of infection (Zbikowska 2005). In the present study, the mitochondrial genome of *T. szidati* from China was sequenced. Its genetic characteristics and phylogenetic status was assessed in order to provide useful information for further study on trematode evolution.

*Trichobilharzia szidati* cercariae were collected from host snail *Lymnaea stagnalis* in Henan, China. (34°40′E; 112°21′N) and was stored in Henan Agricultural University (No. Tsz002). Total genomic DNA was isolated from this specimen according to Webster et al (Webster et al. 2007), and was stored in the sequencing company (BGI Tech, Shenzhen, China). We constructed sequencing libraries using a NEB Next Ultra II DNA Library Prep Kit (NEB, Beijing, China) following the manufacturer’s instructions. Whole genomic sequencing was performed using an Illumina HiSeq 2500 Platform (Illumina, San Diego, CA, USA). The mitogenome was assembled and annotated as reported (Li, Liao, et al. 2018; Li, Wang, et al. 2018; Li et al. 2019; Li et al. 2019a).

The complete mitochondrial genome of *T. szidati* is 14, 303 bp in length and consists of 3023 (21.1%) adenine, 1153 (8.1%) cytosine, 3432 (24.0%) guanosine and 6695 (46.8%) thymine. The genome contains 12 conserved core protein-coding genes (*atp6, cox1, cox2, cox3, nad1, nad2, nad3, nad4, nad4L, nad5, nad6, CYTB*), 21 tRNA genes, 2 rRNA genes and 1 D-loop region. The *T. szidati* mitochondrial genome sequence was submitted to GenBank under the accession number of MG570047.

We used maximum likelihood (ML) and Bayesian inference (BI) to create phylogenies based on the combined gene alignment (Li, Yang, et al. 2018; Li et al. 2019b; Li, Ren, et al. 2019). The ML analysis was performed with RAxML (Stamatakis 2014), while bootstrap values were calculated using 1,000 replicates to assess node support (Li, He, et al. 2020). Bayesian analyses were performed with MrBayes v3.2.6 (Ronquist et al. 2012). Phylogenetic analysis showed that *T. szidati* has a close relationship with *T. regent* (Semyenova et al. 2017) (Figure 1).

**Figure 1.**
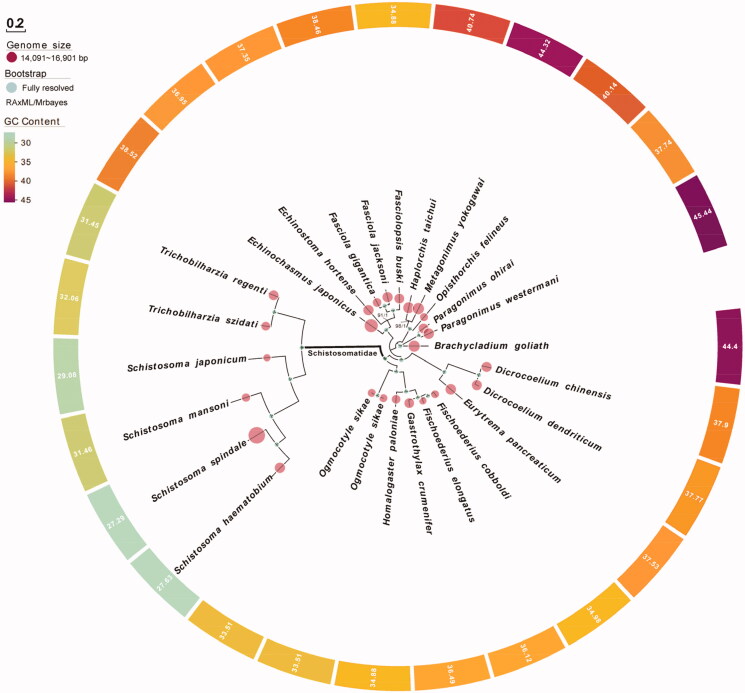
Phylogenetic relationships of 26 digeneans based on Bayesian inference and maximum likelihood analysis of 12 protein-coding genes. Support values are bootstrap values (before slash) and bayesian posterior probabilities (after slash). *Brachycladium goliath* (KR703278); *Dicrocoelium chinensis* (KF318786); *Dicrocoelium dendriticum* (KF318787); *Echinochasmus japonicas* (KP844722); *Echinostoma hortense* (KR062182); *Eurytrema pancreaticum* (KP241855); *Fasciola gigantica* (KF543342); *Fasciola jacksoni* (KX787886); *Fasciolopsis buski* (KX169163); *Fischoederius cobboldi* (KX169164); *Fischoederius elongates* (KM397348); *Gastrothylax crumenifer* (KM400624); *Haplorchis taichui* (KF214770); *Homalogaster paloniae* (KT266674); Metagonimus yokogawai (KC330755); *Ogmocotyle sikae* 1 (KR006934); *Ogmocotyle sikae* 2 (NC_027112); *Opisthorchis felineus* (EU921260.2); *Paragonimus ohirai* (KX765277); *Paragonimus westermani* (KX943544); *Schistosoma haematobium* (DQ157222.2); *Schistosoma japonicum* (JQ781206); *Schistosoma mansoni* (NC_002545); *Schistosoma spindale* (DQ157223); *Trichobilharzia regent* (DQ859919).

## References

[CIT0001] Brant SV, Loker ES. 2009. Molecular systematics of the avian schistosome genus *Trichobilharzia* (Trematoda: Schistosomatidae) in North America. J Parasitol. 95(4):941–963. 20049999 10.1645/GE-1870.1 PMC2922959

[CIT0010] Kašný M, Mikeš L, Doleckova K, Hampl V, Dvorak J, Novotný M, Horak P. 2011. Cathepsins B1 and B2 OF *Trichobilharzia* SPP., bird schistosomes causing cercarial dermatitis. Adv Exp Med Biol. 712:136–154. 21660663 10.1007/978-1-4419-8414-2_9

[CIT0002] Le TH, Blair D, McManus DP. 2002. Mitochondrial genomes of parasitic flatworms. Trends Parasitol. 18(5):7. 10.1016/s1471-4922(02)02252-3 11983601

[CIT0017] Li Q, He X, Ren Y, Xiong C, Jin X, Peng L, Huang W. 2020. Comparative mitogenome analysis reveals mitochondrial genome differentiation in ectomycorrhizal and asymbiotic Amanita species. Front Microbiol. doi: 10.3389/fmicb.2020.01382. PMC7318869 32636830

[CIT0003] Li Q, Liao M, Yang M, Xiong C, Jin X, Chen Z, Huang W. 2018. Characterization of the mitochondrial genomes of three species in the ectomycorrhizal genus Cantharellus and phylogeny of Agaricomycetes. Int J Biol Macromol. 118(Pt A):756–769. 29959010 10.1016/j.ijbiomac.2018.06.129

[CIT0018] Li Q, Ren Y, Shi X, Peng L, Zhao J, Song Y, Zhao G. 2019. Comparative Mitochondrial Genome Analysis of Two Ectomycorrhizal Fungi (Rhizopogon) Reveals Dynamic Changes of Intron and Phylogenetic Relationships of the Subphylum Agaricomycotina. Int J Mol Sci. 20(20):5167. 10.3390/ijms20205167 PMC6829451 31635252

[CIT0004] Li Q, Wang Q, Chen C, Jin X, Chen Z, Xiong C, Li P, Zhao J, Huang W. 2018. Characterization and comparative mitogenomic analysis of six newly sequenced mitochondrial genomes from ectomycorrhizal fungi (Russula) and phylogenetic analysis of the Agaricomycetes. Int J Biol Macromol. 119:792–802. 10.1016/j.ijbiomac.2018.07.197 30076929

[CIT0005] Li Q, Wang Q, Jin X, Chen Z, Xiong C, Li P, Liu Q, Huang W. 2019. Characterization and comparative analysis of six complete mitochondrial genomes from ectomycorrhizal fungi of the Lactarius genus and phylogenetic analysis of the Agaricomycetes. Int J Biol Macromol. 121:249–260. 30308282 10.1016/j.ijbiomac.2018.10.029

[CIT0006] Li Q, Wang Q, Jin X, Chen Z, Xiong C, Li P, Zhao J, Huang W. 2019a. Characterization and comparison of the mitochondrial genomes from two Lyophyllum fungal species and insights into phylogeny of Agaricomycetes. Int J Biol Macromol. 121:364–372. 30315880 10.1016/j.ijbiomac.2018.10.037

[CIT0007] Li Q, Wang Q, Jin X, Chen Z, Xiong C, Li P, Zhao J, Huang W. 2019b. The first complete mitochondrial genome from the family Hygrophoraceae (Hygrophorus russula) by next-generation sequencing and phylogenetic implications. International Journal of Biological Macromolecules. 122:1313–1320. 30227210 10.1016/j.ijbiomac.2018.09.091

[CIT0008] Li Q, Yang M, Chen C, Xiong C, Jin X, Pu Z, Huang W. 2018. Characterization and phylogenetic analysis of the complete mitochondrial genome of the medicinal fungus Laetiporus sulphureus. Sci Rep. 8(1):9104. 29904057 10.1038/s41598-018-27489-9 PMC6002367

[CIT0009] Loker S. 2009. Molecular systematics of the avian schistosome genus *Trichobilharzia* (Trematoda: Schistosomatidae) in North America. J Parasitol. 95(4):941–963. 20049999 10.1645/GE-1870.1 PMC2922959

[CIT0011] Ronquist F, Teslenko M, van der Mark P, Ayres DL, Darling A, Hohna S, Larget B, Liu L, Suchard MA, Huelsenbeck JP. 2012. MrBayes 3.2: efficient Bayesian phylogenetic inference and model choice across a large model space. Systematic Biology. 61(3):539–542. 22357727 10.1093/sysbio/sys029 PMC3329765

[CIT0012] Semyenova S, Chrisanfova G, Mozharovskaya L, Guliaev A, Ryskov A. 2017. The complete mitochondrial genome of the causative agent of the human cercarial dermatitis, the visceral bird schistosome species *Trichobilharzia szidati* (platyhelminthes: Trematoda: Schistosomatidae). Mitochondrial DNA Part B. 2(2):469–470. 33473866 10.1080/23802359.2017.1347833 PMC7800666

[CIT0013] Stamatakis A. 2014. RAxML version 8: a tool for phylogenetic analysis and post-analysis of large phylogenies. Bioinformatics 30(9):1312–1313. 24451623 10.1093/bioinformatics/btu033 PMC3998144

[CIT0014] Webster BL, Rudolfova J, Horak P, Littlewood DT. 2007. The complete mitochondrial genome of the bird schistosome *Trichobilharzia regenti* (Platyhelminthes: Digenea), causative agent of cercarial dermatitis. J Parasitol. 93(3):553–561. 17626347 10.1645/GE-1072R.1

[CIT0015] Yakhchali M, Hosseinpanahi A, Malekzadeh-Viayeh R. 2016. Molecular evidence of trichobilharzia species (Digenea: Schistosomatidae) in the snails of *Lymnaea auricularia* from Urmia Suburb, North West Iran. Iran J Parasitol. 11(3):296–302. 28127334 PMC5256045

[CIT0016] Zbikowska E. 2005. Do larvae of *Trichobilharzia szidati* and echinostoma revolutum generate behavioral fever in *Lymnaea stagnalis* individuals? Parasitol Res. 97(1):68–72. 15952039 10.1007/s00436-005-1394-7

